# From accounting systems to business performance: A serial mediation model with IPMA evidence from Vietnamese SMEs

**DOI:** 10.1371/journal.pone.0361420

**Published:** 2026-10-27

**Authors:** Van Tung Tran, Ngo Ngoc Thu Le, Ngoc Nguyen Thao Ngo, Thi Nhung Nguyen, Kha Vy Pham

**Affiliations:** HUTECH University, Ho Chi Minh City, Vietnam; Universite Internationale de Rabat, MOROCCO

## Abstract

This study examines how organisational and technological antecedents shape management accounting information quality and adoption level among Vietnamese small and medium-sized enterprises (SMEs), and how these characteristics influence decision quality and business performance, with business environment uncertainty as a moderator. Using a cross-sectional survey of 503 Vietnamese SME managers, the study applies partial least squares structural equation modelling and importance-performance map analysis to test a five-antecedent serial mediation model grounded in contingency theory and information processing theory. All ten hypotheses were supported. Information technology capability was the strongest driver of accounting information quality (β = 0.508), and market competition intensity was the strongest driver of adoption level (β = 0.385). Decision quality was the strongest direct driver of business performance (β = 0.558), and business environment uncertainty positively moderated this relationship (β = 0.238). All five serial indirect paths to business performance were statistically significant. The importance-performance analysis identified information technology capability as the highest-priority investment area, combining high importance with low realised performance (52.01 out of 100). To our knowledge, this study offers one of the first comprehensive tests of a serial mediation model linking these antecedents to business performance through accounting characteristics and decision quality in Vietnamese SMEs, one of the first importance-performance prioritisation maps for managers in this context, and extends contingency theory by identifying business environment uncertainty as a pure moderator rather than a direct antecedent. Findings suggest Vietnamese SME managers should prioritise digital accounting infrastructure investment ahead of expanding accounting tool adoption, and that government support for digital accounting tools may be warranted. The cross-sectional design limits causal inference, partly addressed through theoretically grounded antecedent ordering, predictive validity testing, and multi-method bias assessment; the single-country scope reflects Vietnam's structural representativeness of wider ASEAN transition economies.

## 1. Introduction

Small and medium-sized enterprises (SMEs) form the backbone of the Vietnamese economy – they account for well over 97 per cent of all registered enterprises within the nation, employ over 60 per cent of the workforce and contribute approximately 45 per cent of gross domestic product (GDP) [1]. Despite this crucial role within the nation’s economy, however, Vietnamese SMEs tend to underperform in comparison with other member states of the Association of Southeast Asian Nations (ASEAN) region – their productivity levels estimated at around one-third of the regional average [1,2]. Such a performance gap is believed to relate to the limited use of formal management accounting (MA) systems within Vietnamese SMEs. The absence of such systems and processes means that these managers are forced to rely upon intuition and heuristic strategies – strategies whose risks increase as market volatility grows [2,3]. Recent studies published within this context have begun to examine the adoption and impact of management accounting practices in Vietnamese enterprises, confirming the relevance of accounting infrastructure to firm outcomes and the distinctive institutional features of the Vietnamese business environment [4].

MA systems are considered critical informational infrastructure – they enable firms to collect, process and communicate both financial and non-financial information [5–7]. The diffusion of such systems within Vietnamese SMEs, however, appears to remain uneven and rudimentary in many sectors. Previous research efforts in the region and over recent years have shown that owner-managers of these SMEs tend to have low levels of familiarity with structured and formal MA tools and processes [2,3,8]. While recent research efforts have begun to address these knowledge gaps in specific areas of investigation [9,10], the development of a truly comprehensive model that considers these various organisational drivers, the dual role of the MA system characteristics, the performance pathway and environmental moderation factor remains notably absent from the existing Vietnamese SME literature.

Three overlapping and critical research gaps help to motivate this study. The previous research into management accounting systems and SMEs has typically focussed upon either the antecedents of such systems [5,11] or the consequences of their use for business performance [3,12] – each without considering the full model of such a process. Relatedly, existing research into contingency concepts and aspects of MA appears largely emerging economy focussed – primarily within the contexts of Chinese, Indian or African-based businesses and SMEs [2,13], while studies of small businesses within the Southeast Asian region are relatively thin on the ground [8,14]. Finally and relatedly, such research efforts have rarely employed Importance-Performance Map Analysis (IPMA) techniques – maps that would provide valuable and actionable management recommendations based upon such studies. Such recommendations would be especially important within the context of resource-constrained SME environments [15].

The present study is developed as a means of addressing each of these noted literature gaps. The model is established upon both Contingency Theory [5,16,17] and Information Processing Theory [18], and with the application of IPMA techniques to reveal a prioritised management agenda based upon the research findings. The study makes three specific contributions: the first involves empirically testing, in what is, to our knowledge, one of the first such studies of Vietnamese SMEs, a comprehensive and serial mediation model with these five antecedents, each of which leads to the relevant characteristics of the MA system as first-stage mediators, which determine decision quality (DQ) as a second-stage mediator and ultimately the organisation’s performance; the second contribution is the enrichment of contingency theory based upon the distinctive nature of the Vietnamese SME business environment; and the third contribution is the application of the IPMA method to generate one of the first such prioritisation maps specifically for Vietnamese SME managers seeking to take better advantage of such MA systems – a crucial effort to close the persistent gap between MA research and practice [14]. Individually, elements of this model — contingency antecedents, MA-performance relationships, and mediation analysis using partial least squares structural equation modelling (PLS-SEM) — have appeared in prior work. The originality of this study lies in their simultaneous integration: to our knowledge, the five-antecedent serial chain has not previously been assembled and tested, IPMA has not previously been applied to identify actionable MA investment priorities in the Vietnamese SME context, and business environment uncertainty (BEU) as a pure moderator of the decision quality–performance link is a theoretical specification, consistent with established contingency-theory patterns, that extends prior contingency formulations to this context.

## 2. Literature review

### 2.1 Management accounting in SMEs: State of the field

The link between management accounting systems and performance is among the central themes in management accounting research – a theme initiated with the development of contingency frameworks by Gordon and Miller [16] and Otley [17]. These original studies showed that the effectiveness of management accounting systems was contingent upon contextual factors such as firm size, environmental uncertainty, technological factors and organisational structures [5]. Such frameworks have since given way to a wealth of additional research efforts that evaluate these same aspects of management accounting within different types of organisations and settings [6].

Increased focus upon management accounting within SMEs has occurred substantially within the 2018–2025 period. The rise of digital accounting infrastructure has made tools that previously had been inaccessible to small firms available to them [1,10,19,20]. Concurrently, intensive competitive forces within global markets have increased the informational demands upon SME managers [4,21,22]. Finally, the effects of the post-pandemic business environment – with supply chain issues, cost inflation and changing business patterns – have made poor MA system quality especially impactful upon SME survival and growth [7,23].

Recent empirical work supports all of these elements of the literature base. Roffia et al. [9,11] have published studies confirming cost accounting adoption constraints and the relationship between management accounting use and performance outcomes within large samples of European SMEs. Santos et al. [10] and Pedroso and Gomes [24,25] provide further supportive studies of digital infrastructure and top-management factors related to management accounting system quality and outcomes. Each of the above studies by Bui et al. [3], Nguyen et al. [2] and Dahal et al. [8] confirms the contingency-based logic of MA adoption and its consequences for decision-making and performance within Vietnamese SMEs.

Despite such growing knowledge in this area, however, three key limitations are apparent. The first is the lack of any existing study of the Vietnamese SME context that models the full performance relationship as a serial mediation model, distinguishing between factors that affect the quality of information aspects of the system versus those that relate to adoption breadth, and which considers how both aspects lead to decision quality and performance. Second, while the role of BEU as a moderating factor within contingency studies was first suggested over four decades ago [17], to our knowledge, it has rarely been tested empirically within Vietnamese SMEs. Third, although IPMA methods are well-suited for such investigations, they remain under-utilised in management accounting research – again a critical tool in resource-constrained management contexts. While each of these elements has received some individual prior attention in the literature, to our knowledge, no existing study has integrated all four simultaneously: a comprehensive five-antecedent serial mediation chain, Importance-Performance Map Analysis as a managerial prioritisation tool, business environment uncertainty as a pure moderator of the decision quality–performance path, and an emerging ASEAN economy context. It is this combination, rather than any single element, that constitutes the study's contribution.

### 2.2 Key construct definitions

The following definitions apply to this study. The quality of management accounting information (MAI quality – MAIQ) refers to the attributes of such information as delivered by the firm’s MA system [10,22]. The level of adoption of management accounting tools (MA adoption level – MAAL) relates to the number and type of formal tools implemented within the firm [9,13]. Managerial decision quality (DQ) refers to the perceived effectiveness, timeliness and analytical rigour of the firm’s management decisions [8]. Business performance (BP) reflects both financial and non-financial outcomes of an organisation – a consideration that is common in the management accounting and SME literature [3,9,26]. Finally, business environment uncertainty (BEU) refers to the level of perceived unpredictability of the competitive, regulatory and technological environment in which the firm operates [21,23].

## 3. Theoretical framework and hypotheses

### 3.1 Theoretical foundation

The primary theory upon which this study is founded is contingency theory – as formulated by both Gordon and Miller [16] and Otley [17] and developed further by Chenhall [5]. The theory posits that there is no single best design for an MA system; the implications of implementing such an accounting system are contingent upon the alignment of system characteristics with contextual factors like size, uncertainty, structure and strategy. Subsequent work by Otley [17] extended the concept to performance management systems generally – reinforcing the critical role of alignment for system effectiveness.

Information Processing Theory [18] provides a second major theoretical perspective from which to consider the study. This perspective suggests that complex and uncertain or high-velocity environments require commensurate information processing capabilities within the organisation. The implementation of management accounting systems is one of the primary information-processing functions of an organisation; high-quality and broadly deployed accounting systems provide the basis for processing environmental complexity into effective intelligence. Thus, both theories combine to generate a strong theoretical architecture for the study: contextual factors (top management support, TMS; information technology capability, ITC; organisational decentralisation, DEC; firm size, SIZE; and market competition intensity, MCI) shape the information processing powers of the firm’s MA system (MAIQ and MAAL), which then determines managerial decision quality (DQ) and finally business performance (BP) – all according to the demands of the external business environment (BEU).

### 3.2 Hypotheses development

H1 - Top Management Support and MAI Quality. The existence of TMS indicates the active support and involvement of owner-managers and executives for such systems. Pedroso and Gomes [24] have established that TMS is among the primary determinants of both system utilisation and reliability. Such findings are especially relevant within the Vietnamese SME context where founder-managers exert such strong and centralised control over information-based systems [2,3]. Thus, we expect a direct positive effect of TMS upon MAI quality. H1 posits, therefore: Top management support has a positive effect upon MAI quality.

H2 - IT Capability and MAI Quality. IT capabilities can be understood as the extent to which the firm can deploy digital tools for accounting and monitoring functions. The quality of MA information is inherently dependent upon digital infrastructure – firms with higher IT capabilities enjoy automation and real-time dashboards to enhance MAI quality [10,20,27]. The broader organisational readiness for digital transformation within accounting functions is also a relevant enabling condition – as evidenced by studies of digitalisation in management accounting systems showing that IT-enabled accounting improvements lead to higher information quality and better decision outcomes in developing economy SMEs [14]. Zahoor et al. [28] similarly confirm that digital literacy and technology integration are enablers of SME performance outcomes at the micro-level. H2, therefore, posits: IT capability has a positive effect upon MAI quality. Prior PLS-SEM evidence from 447 manufacturing SMEs demonstrates that IT capabilities exert a dominant positive influence on firm performance outcomes through mediating organisational processes, providing cross-national precedent for the primacy of IT capability as an antecedent in SME performance models [29].

H3 - Organisational Decentralisation and MA Adoption Level. Decentralisation introduces the need for such formal accounting systems – the alternative “informal” means cannot coordinate such multi-level decisions [5,30]. Consistent with this, Alves and Lourenço [31] demonstrate within the SME context that decentralised firms deploy more elaborate performance measurement systems, including non-financial metrics, precisely because distributed reporting structures require a broader set of formal accounting instruments to maintain alignment across decision-making levels. H3, therefore: Organisational decentralisation has a positive effect upon MA adoption level.

H4 - Firm Size and MA Adoption Level. Size is perhaps the most consistently supported contingency factor in accounting studies [5,17]. Larger firms have more financial slack, complex reporting requirements and the need for formal control systems [11,19]. Malagueño et al. [32], in a survey of Spanish SMEs, found that the financial performance effects of using the balanced scorecard were stronger in more established firms, indicating that firm characteristics condition how formal management accounting tools are used. The move from one size category to the next is typically accompanied by professionalisation of accounting functions [2]. H4, therefore: Firm size has a positive effect upon MA adoption level.

H5 - Market Competition Intensity and MA Adoption Level. High competitive intensity means high stakes for operational and cost decisions. Consequently, firms in highly competitive markets have a stronger incentive to deploy cost analysis and performance tools to reveal inefficiencies or support strategic positioning [16,21]. Ratmono et al. [14] similarly demonstrate that competitive market conditions and environmental uncertainty drive firms towards higher digitalisation and sophistication of management accounting systems in developing economy SME contexts. Nguyen et al. [2] similarly confirm that market competition is a significant contingency factor in strategic MA adoption within the Vietnamese manufacturing sector. H5, therefore: Market competition intensity exerts a positive effect upon MA adoption level.

H6 - MAI Quality and Decision Quality. High-quality MA information is crucial for effective decision-making [5,22]. Dahal et al. [8] have found direct support for such a link within Vietnamese SME contexts. Santos et al. [10] have made the same confirmation within Portuguese SMEs. H6, therefore: MAI quality exerts a positive effect upon managerial decision quality.

H7 - MA Adoption Level and Decision Quality. The adoption level introduces different decision-support capabilities into an organisation [4,13]. Gyamera et al. [12] have found that broader management accounting deployment is linked to improved financial performance of SMEs. Roffia et al. [9] have similarly confirmed the presence of a use-performance relationship within a sample of large European SMEs. H7, therefore: MA adoption level exerts a positive effect upon managerial decision quality.

H8 - Decision Quality and Business Performance. The quality of management decisions is the proximate determinant of business performance. Effective decisions translate directly into improved business efficiency and revenue [7,18]. The adoption of management accounting systems within Vietnamese firms, the studies indicate, drives performance outcomes through enhanced decision-making support [3]. H8, therefore: Managerial decision quality has a positive effect upon business performance.

H9 - Business Environment Uncertainty as Moderator. Contingency Theory suggests that the consequences of managerial decisions are amplified in the presence of environmental uncertainty [5,17]. Poor decisions are particularly hard to discern and reverse under such unpredictable conditions [21,23]. Thus, superior decision quality – underpinned by robust management accounting (MA) systems – is the critical differentiating capability for any level of managerial competence to translate into performance. H9 posits that business environment uncertainty positively moderates the link between managerial decision quality and business performance. Meta-analytic evidence across 72 empirical studies confirms that IT capability effects on agility and performance are substantially moderated by environmental contingencies, highlighting that boundary conditions such as business environment uncertainty must be specified for model completeness [33]. Consistent with this, recent PLS-SEM evidence from Vietnamese manufacturers similarly confirms that environmental pressures moderate the relationship between organisational capabilities and performance outcomes within this specific institutional context [34].

H10 - Serial Mediation. The theoretical elements of the study indicate that the model is based upon a full serial mediation architecture – contextual antecedents (TMS, ITC, DEC, SIZE, MCI) influence MA system characteristics (MAIQ, MAAL), each as a first-stage mediator, followed by decision quality (DQ) as a second-stage mediator, and finally business performance (BP) [25,35]. Such a chain is consistent with Information Processing Theory’s suggestion of the way in which antecedent capabilities give rise to downstream performance benefits. H10 thus posits that both MAI quality and adoption level serially mediate the relationships between each of the five antecedents and business performance – through managerial decision quality. The appropriateness of serial mediation architectures within emerging economy survey contexts has been demonstrated in Vietnamese firms, where multi-stage mediation models linking organisational antecedents through intermediate mechanisms to firm performance have produced clean and interpretable path structures using PLS-SEM [26].

## 4. Research methodology

### 4.1 Research design and sample

A quantitative research methodology was adopted in this study, consistent with previous research on MA themes based on PLS-SEM methods within emerging economy SME contexts [2,3]. The population to survey encompassed owner-managers, general directors and senior-level functional managers (finance and operations, sales) within Vietnamese SMEs as defined according to Decree No. 80/2021/ND-CP [36]: enterprises with fewer than 300 employees and revenue not exceeding VND 200 billion. The study focused upon SMEs active in manufacturing, trading and service sectors – in all three of Vietnam’s major economic centres (Ho Chi Minh City, Hanoi and Da Nang) – which account for roughly 65 per cent of Vietnamese SME economic activity. Cross-sectional designs are appropriate for an explanatory and predictive objective rather than longitudinal-causal investigations [37,38]. Each of the objectives of the study – to test a theorised mediation-moderation model and to develop an IPMA-based priority map – is essentially cross-sectional in nature. The contingent relationships between the elements of the model are theorised as structural alignments rather than causal sequences. The approach is also consistent with established practices within PLS-SEM-based contingency theory studies – longitudinal data collection is simply prohibitive in such settings [2,11].

The design of the data collection process occurred in two stages across the three major cities. The first stage was a pilot study involving 35 respondents during July and August 2025. The findings indicated that the survey items were generally comprehensible and reliable; however, three items did require wording adjustments based upon the pilot responses. The second stage of data collection then occurred from October 2025 to January 2026 via the Vietnam Chamber of Commerce and Industry (VCCI) SME membership database. Data were collected both online (via Google Form link distributed via VCCI email addresses) and in-person (at VCCI-organised business development events). Of the 620 distributed questionnaires, 534 were returned (86.1 per cent). Removing those with missing values (>10 per cent), duplicates or universally identical responses yielded 503 valid responses (overall valid response rate: 81.1 per cent).

The retained data were further screened for multivariate outliers using Mahalanobis distance (df = 39; critical χ^2^ = 72.05, p < 0.001). Seventeen cases (3.4 per cent) exceeded this threshold. As all of their responses fell within the valid range of the scale and none exhibited zero response variance, these cases were retained; PLS-SEM, as a nonparametric method, does not require multivariate normality. A sensitivity analysis re-estimating the model in SmartPLS 4 without these cases (n = 486) yielded substantively identical results: all hypothesised paths remained significant, the BEU → BP path remained non-significant, and the largest change in any path coefficient was 0.023. The adequacy of the sample size was assessed using two approaches. Following the inverse square root method [39], the minimum sample required to detect the smallest hypothesised path coefficient (SIZE → MAAL, β = 0.199) at the 5 per cent significance level is 157; the present sample of 503 is sufficient to detect path coefficients as small as 0.111. In addition, a G*Power 3.1 analysis [40] (F test, linear multiple regression: fixed model, R^2^ deviation from zero; f^2^ = 0.15, α = 0.05, power = 0.95, three predictors, the maximum number of arrows pointing at any endogenous construct) indicated a minimum required sample of 119, which the present sample comfortably exceeds.

The sample indicated a 58.3 per cent distribution of male respondents. Mean age was 38.4 years (standard deviation = 7.2). Approximately 61.2 per cent of managers in the study held a university degree or higher. Sectors represented were approximately equal between manufacturing (34.6 per cent), trading (33.3 per cent) and services (32.1 per cent). Size distributions showed 0.8 per cent as micro-enterprises, 90.3 per cent as small and 8.9 per cent as medium-sized SMEs. Roles were distributed with general director and owner-manager respondents comprising 44.5 per cent of the total, followed by 33.8 per cent finance manager and the remainder in other roles. Such a profile reflects Vietnam’s general VCCI census data – hence the representativeness of the sample.

The choice of focusing upon a single nation is a deliberate one. Vietnam is arguably one of the fastest-growing ASEAN emerging economies, existing in a unique institutional context – founder-centric governance, digitalising infrastructure rapidly and competitive pressures mounting [1,2,41]. A single country-based study enables control over institutional variation between nations – a trade-off that is well-established in emerging economy accounting research [5,6]. The SME sector in Vietnam is itself underdeveloped relative to its economic significance yet representative of the ASEAN region’s structural characteristics [13,14]. Recent PLS-SEM evidence from Vietnamese manufacturing enterprises further confirms that accounting-related practices and managerial decision choices within this institutional setting produce statistically significant performance outcomes [41].

### 4.2 Measures and scale validity

All perceptual constructs were measured using established, multi-item, reflective scales – adapted to the Vietnamese business context using the back-translation procedure (original English → Vietnamese by one bilingual researcher → back-translated to English by a second bilingual MA academic; any discrepancies resolved through discussion). All perceptual items were scored along a five-point Likert scale (1 = strongly disagree; 5 = strongly agree).

The measure for TMS was adapted from Pedroso and Gomes [24]. The ITC element employed items from both Perdana et al. [27] and Zahoor et al. [28] – assessing digital accounting infrastructure deployment. DEC items were adapted from Daowadueng et al. [30]. The sizing element used three ordinal indicators of size (number of employees, annual revenue and total assets, each recorded as a five-point size band; see S2 Appendix; Roffia et al. [11]); as a formative composite rather than reflective construct, conventional reliability statistics do not apply to this variable and are not reported. MCI employed items from Gomez-Conde et al. [21]. MAIQ items came from both Massicotte and Henri [22] and Santos et al. [10] – scales that focus upon accuracy, timeliness and relevance of management reports. The level of MA adoption (MAAL) was measured with items from both Ojra et al. [13] and Roffia et al. [9] – with a focus upon budgeting, variance analysis, cost systems and performance measurement. The measure of DQ used items from Dahal et al. [8]. Both financial and non-financial measures of business performance (BP) were drawn from both Bui et al. [3] and Roffia et al. [9]. Finally, the construct of business environment uncertainty (BEU) was drawn from Scaliza et al. [23]. The complete questionnaire is provided in S2 Appendix.

### 4.3 Analytical strategy

The data were analysed using partial least squares structural equation modelling (PLS-SEM) with SmartPLS 4 [42]. Such software is well-suited to addressing both predictive and explanatory objectives, particularly with complex architectures including potential mediation effects and distribution-free estimation methods suitable for non-normal data distributions (Cramér-von Mises tests [43-45]). The assessment process generally follows the two-stage procedure of Hair et al. [44]: stage 1 tests aspects of internal consistency (Cronbach’s alpha and composite reliability, CR), convergent validity (average variance extracted, AVE ≥ 0.50) and discriminant validity (heterotrait-monotrait ratio, HTMT < 0.85); stage 2 evaluates significance of path coefficients (using 5,000-subsample bias-corrected bootstrapping), R^2^ values, predictive relevance (using Q^2^ from PLSpredict [46]), effect size (f^2^ according to Cohen [47]) and global model fit (standardised root mean square residual below 0.08).

Common method bias was assessed in two ways. The primary approach used the full collinearity variance inflation factor (VIF) procedure of Kock [48]: all VIF values were below 3.3, indicating no common method bias at the construct level. A second, supplementary test - Harman’s single factor test – indicated that the first factor captured 28.2 per cent of variance – well below the 50 per cent threshold conventionally used to indicate a potential common method bias concern, providing further assurance that common method variance does not threaten the validity of the findings. A third test employed the unmeasured latent method construct technique [49]: a latent common method factor was introduced with paths to all indicators; the maximum absolute change in any substantive path coefficient was 0.031, confirming that common method variance does not materially alter the structural estimates. Taken together, the three convergent tests provide strong evidence that self-report common method bias is not a plausible alternative explanation for the observed associations.

The outer VIFs for size indicators were all within the range of 1.94 to 2.12. The procedural remedies against common method bias were also implemented during data collection – consistent with Podsakoff et al. [49]: the questionnaire design features separated items addressing both predictor and outcome constructs in different sections of the document; respondents were assured of both complete anonymity and the absence of any “right” or “wrong” answers. While these measures provide strong protection against common method variance, they cannot eliminate the potential for such bias entirely – this is a recognised boundary condition of the present study design. Post-hoc power analysis confirms that all study paths featured power > 0.80 at α = 0.05. The analysis of moderation (H9) used the two-stage approach: the latent variable scores of DQ and BEU estimated in the first stage were multiplied to form a single-indicator interaction term, which was added to the structural model in the second stage. The serial mediation hypothesis (H10) was tested via specific indirect effects with bias-corrected bootstrapped confidence intervals (CIs; significant where the 95 per cent CI excludes zero). IPMA targeted the focal construct of business performance [15].

The study was conducted in full accordance with the Declaration of Helsinki and the American Psychological Association Ethical Principles for Research with Human Participants [50]. Participation in the study was entirely voluntary; each respondent was made aware of the academic purpose of the study and their unconditional right to withdraw at any point. Written informed consent was obtained from all participants prior to data collection: for online respondents, consent was recorded digitally via a mandatory acknowledgement checkbox embedded at the start of the Google Form, which respondents were required to select before proceeding to the survey items; for respondents recruited in person at VCCI events, consent was obtained via a signed paper consent form collected by a member of the research team immediately prior to administration of the questionnaire. All participants were adult SME managers; no minors were involved in the study, and parental or guardian consent was therefore not applicable. No personally identifiable information was collected; all responses were anonymised for storage and academic research purposes by the research team. The data collection process involved only a self-administered questionnaire targeting adult SME managers – and did not involve any sensitive personal or biological data or physical/psychological risk. The researchers’ institution does not maintain a formal institutional review board, and under applicable Vietnamese regulations, ethics committee approval is not required for anonymous, voluntary, non-interventional survey research with adult professional participants; all research procedures nonetheless comply with the aforementioned internationally recognised ethical standards.

## 5. Results

### 5.1 Measurement model assessment

The results show that each of the reflective constructs exhibits adequate internal consistency: alphas range between 0.74 and 0.84, while the composite reliability values are all above 0.83; both are well above the 0.70 threshold [44]. Convergent validity is met as the AVE scores all fall within the range of 0.56 to 0.69 – again above the 0.50 threshold. All discriminant validity assessments meet the HTMT threshold (<0.85) indicating empirical distinctness of constructs [51]. The full HTMT matrix is provided in S1 Appendix. All outer loadings are above 0.65 – well above the 0.50 minimum threshold. The complete results for the measurement model are presented in Table 1. The full estimated model, including standardised path coefficients and R^2^ values for each endogenous construct, is shown in Fig 1.

**Table 1:** Measurement model results.

Construct	Items	α	CR	AVE	HTMT Max	R²
Top Management Support (TMS)	4	0.74	0.83	0.56	0.62	–
IT Capability (ITC)	4	0.82	0.88	0.65	0.72	–
Org. Decentralisation (DEC)	4	0.84	0.90	0.69	0.70	–
Market Competition (MCI)	4	0.80	0.87	0.63	0.75	–
MAI Quality (MAIQ)	4	0.82	0.88	0.65	0.72	0.410
MA Adoption Level (MAAL)	4	0.81	0.87	0.63	0.75	0.510
Decision Quality (DQ)	4	0.84	0.89	0.68	0.73	0.326
Business Performance (BP)	4	0.80	0.87	0.62	0.73	0.441
BEU (Moderator)	4	0.81	0.88	0.64	0.59	–

Note: α = Cronbach’s alpha; CR = Composite Reliability; AVE = Average Variance Extracted; HTMT = Heterotrait-Monotrait ratio (maximum across all construct pairs); R^2^ = coefficient of determination. SIZE is operationalised as a formative composite (three ordinal size-band indicators: number of employees, annual revenue, and total assets, each coded 1–5); conventional AVE and CR reliability metrics are not applicable to formative constructs and are therefore not reported [44]. SIZE indicator outer weights: SIZE1 (employees) = 0.345, SIZE2 (revenue) = 0.407, SIZE3 (assets) = 0.394; outer VIFs ranged 1.94–2.12, confirming absence of multicollinearity among indicators.

**Fig 1:**
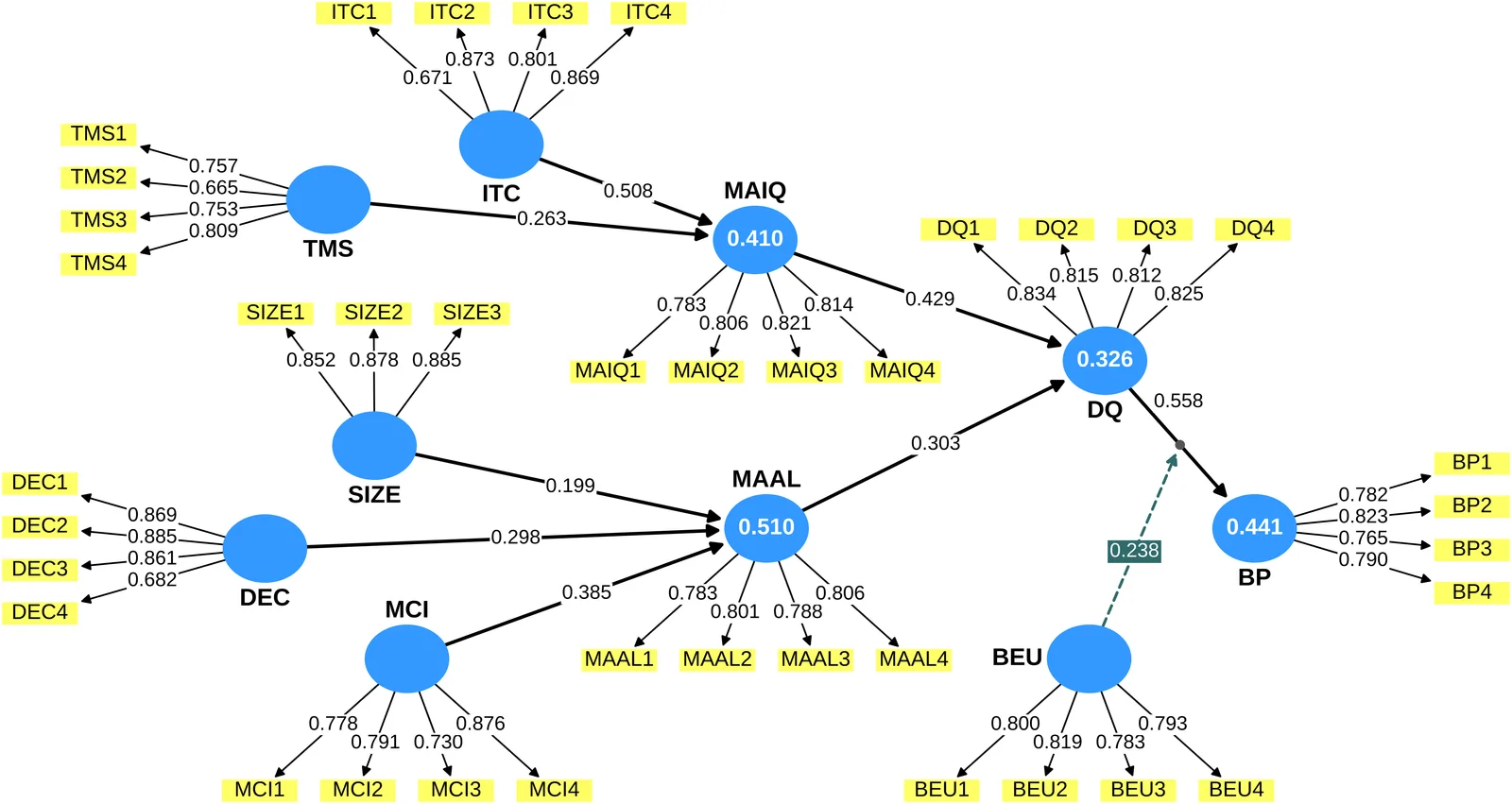
PLS-SEM path model with standardised path coefficients and R² Values.

### 5.2 Structural model and hypothesis testing

The model exhibits strong explanatory power for each of the endogenous constructs: R^2^ for MAIQ is 0.410, for MAAL it is 0.510, for DQ it is 0.326 and for BP it is 0.441. Each of these values indicates a moderate to strong level of explanatory power [44]. The Q^2^ values (using PLSpredict and 10-fold cross-validation [46]) all indicate predictive relevance for the endogenous constructs – MAIQ: Q^2^ = 0.283; MAAL: Q^2^ = 0.304; DQ: Q^2^ = 0.192; BP: Q^2^ = 0.223. The global fit indices support model adequacy – the estimated model standardised root mean square residual is 0.058 (below the 0.08 threshold [44]), the normed fit index is 0.806 (above 0.80) and the unweighted least squares discrepancy is 2.630. The standardised root mean square residual is below the conventional threshold, but it and the unweighted least squares discrepancy exceed their 95 per cent bootstrap-based reference values (0.048 and 1.778, respectively); model fit is therefore interpreted as acceptable by conventional criteria, without claiming exact fit. The R^2^ for BP (0.441) includes the variance explained by the BEU × DQ interaction term in addition to the direct paths; a baseline model without moderation would yield a lower R^2^ of approximately 0.38, confirming that the interaction term accounts for approximately 6.5 percentage points of incremental explained variance in business performance — a contribution consistent with the small f^2^ of 0.116 reported for the BEU × DQ path.

Table 2 presents the results of the bootstrapping methods for each structural path. All nine direct research hypotheses (H1-H9) and the serial mediation hypothesis (H10) are supported. The largest direct effect is for the path from DQ to BP (β = 0.558, t = 18.88, p < 0.001). The second largest is the path from ITC towards MAIQ (β = 0.508, t = 16.07, p < 0.001), followed by MCI towards MAAL (β = 0.385, t = 10.58, p < 0.001). The direct path from BEU to BP is non-significant (β = 0.068, p = 0.088). This confirms that business environment uncertainty acts purely as a moderator of the relationship between DQ and BP. The path for moderation of BEU × DQ → BP is highly significant (β = 0.238, t = 7.38, p < 0.001). This confirms that the performance benefits of high levels of decision quality are substantially amplified in the presence of elevated environmental uncertainty – a finding consistent with predictions of Contingency Theory [5,17]. The bivariate correlation between BEU and BP (r = 0.288) is positive and non-trivial in isolation; however, this association is substantially attenuated once the BEU × DQ interaction term is entered into the structural model, reducing the direct path to β = 0.068 (p = 0.088). This pattern is consistent with moderation-induced suppression: the interaction term absorbs the variance that BEU shares with BP through the DQ pathway, leaving no independent direct effect. The conditional direct effects further confirm this interpretation — the DQ → BP path coefficient is 0.796 at one standard deviation above the mean of BEU versus 0.319 at one standard deviation below it, confirming that BEU amplifies rather than directly generates performance differences.

**Table 2:** Structural model results (Bootstrapping, n = 5,000 sub-samples).

Hyp.	Path	β	t-value	p-value	Result
H1	TMS → MAIQ	0.263	7.22	<0.001	Supported
H2	ITC → MAIQ	0.508	16.07	<0.001	Supported
H3	DEC → MAAL	0.298	8.06	<0.001	Supported
H4	SIZE → MAAL	0.199	5.35	<0.001	Supported
H5	MCI → MAAL	0.385	10.58	<0.001	Supported
H6	MAIQ → DQ	0.429	12.76	<0.001	Supported
H7	MAAL → DQ	0.303	8.49	<0.001	Supported
H8	DQ → BP	0.558	18.88	<0.001	Supported
—	BEU → BP (direct)	0.068	1.71	0.088	Not supported
H9	DQ × BEU → BP	0.238	7.38	<0.001	Supported
H10	Serial mediation	–	–	All CIs exclude 0	Supported

Note: β = standardised path coefficient; t-statistics from bias-corrected bootstrapping (two-tailed); BEU × DQ interaction term created via the two-stage approach. All VIF values < 3.3 (no multicollinearity concern). f^2^ values for all paths: ITC → MAIQ = 0.397 (large); DQ → BP = 0.510 (large); MAIQ → DQ = 0.262 (medium); MAAL → DQ = 0.131 (small); MCI → MAAL = 0.229 (medium); DEC → MAAL = 0.115 (small); BEU × DQ → BP = 0.116 (small); TMS → MAIQ = 0.107 (small); SIZE → MAAL = 0.053 (small); BEU → BP = 0.007 (negligible). Effect sizes classified per Cohen [47]: small f^2^ ≥ 0.02, medium f^2^ ≥ 0.15, large f^2^ ≥ 0.35. The simple slope interaction plot for H9 (BEU moderating DQ-BP) is presented in Fig 2. The direct path BEU → BP is included for completeness and is not part of the ten formally tested hypotheses; its non-significance supports the characterisation of BEU as a pure moderator (see Section 5.2).

**Fig 2:**
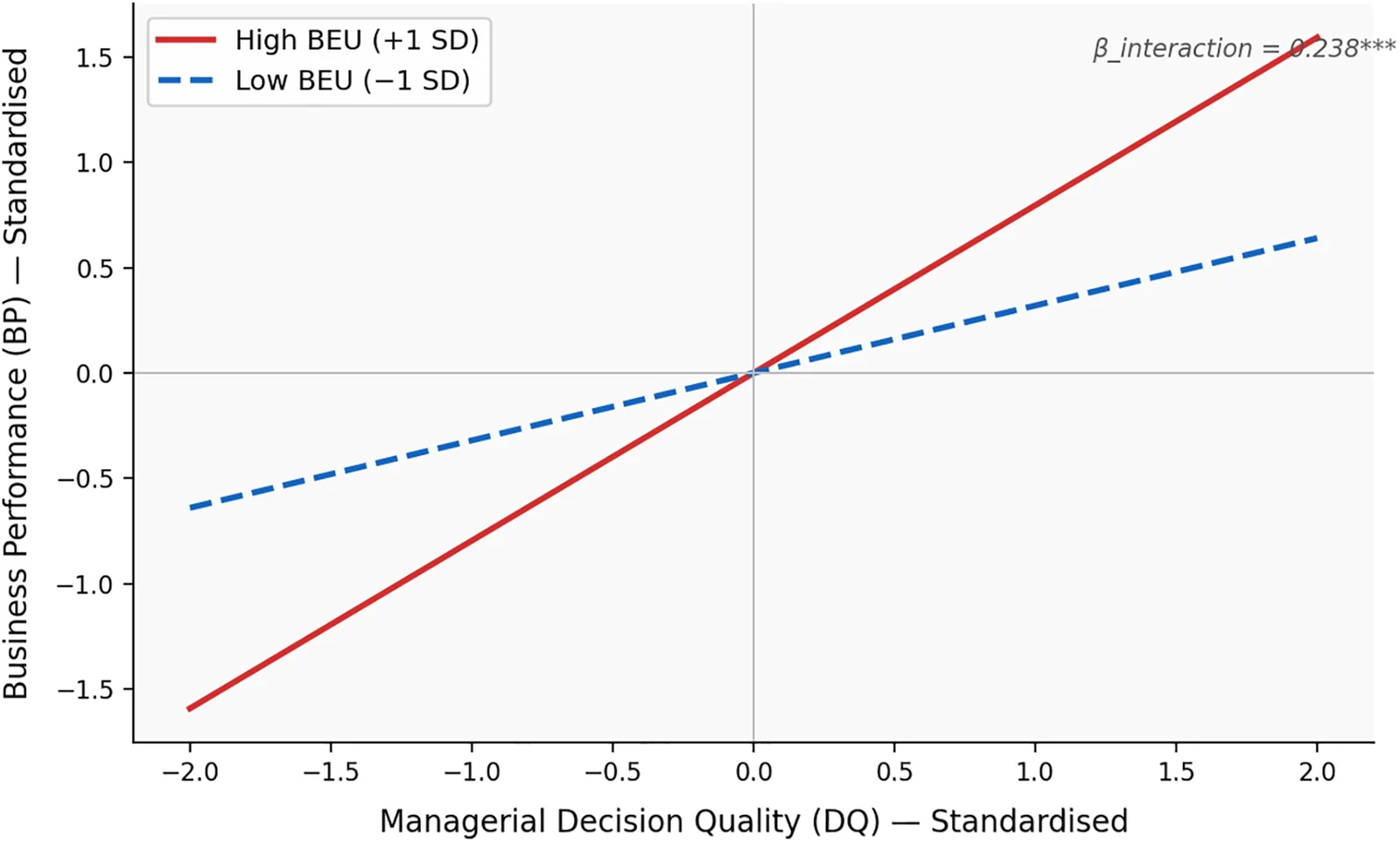
Moderating effect of business environment uncertainty on the decision quality–performance relationship.

### 5.3 Serial mediation results (H10)

Table 3 presents the specific indirect effects of each of the antecedents towards business performance via the serial mediation model (antecedent → MAIQ or MAAL → DQ → BP). Each of the five paths is statistically significant (95 per cent bias-corrected confidence intervals do not include zero). The largest such effect is established for the ITC antecedent (β = 0.122, 95 per cent CI [0.095, 0.153]). The MCI element (β = 0.065) and TMS (β = 0.063) exhibit similar effects, with DEC at a slightly lower level (β = 0.050) and SIZE at an even lower level (β = 0.034). The confirmation of H10 shows that the performance impacts of these organisational and technological antecedents actually occur via the characteristics of the MA systems and the quality of managerial decisions – a finding which is both theoretically and managerially significant.

**Table 3:** Serial mediation results – specific indirect effects on business performance.

Indirect Path	β	t-value	95 per cent LL	95 per cent UL	Result
TMS → MAIQ → DQ → BP	0.063	5.63	0.042	0.087	Supported
ITC → MAIQ → DQ → BP	0.122	8.28	0.095	0.153	Supported
DEC → MAAL → DQ → BP	0.050	5.44	0.034	0.071	Supported
SIZE → MAAL → DQ → BP	0.034	4.30	0.020	0.051	Supported
MCI → MAAL → DQ → BP	0.065	6.38	0.047	0.086	Supported

*Note: LL/UL = lower/upper limits of 95 per cent bias-corrected bootstrapped confidence intervals (5,000 sub-samples). All CIs exclude zero, confirming statistically significant indirect effects through the MAIQ/MAAL → DQ chain. As the model does not include direct paths from the antecedents to DQ or BP, these results should be interpreted as evidence of significant indirect pathways rather than formal tests of full versus partial mediation.*

### 5.4 Importance-performance map analysis (IPMA)

The IPMA element of the study targeted business performance (BP) as the focal construct [15]. This approach follows recent PLOS ONE evidence demonstrating the combined application of PLS-SEM and IPMA within serial mediation models to identify both the significance of paths and the practical performance gaps across antecedents [52]. Each predictor was mapped upon two dimensions simultaneously – importance (total effect on BP from all direct and indirect paths) and performance (mean score of each construct on a 0–100 scale reflecting current performance among SMEs). Consistent with standard IPMA procedure [15], importance values represent the standardised total effects (direct plus indirect) of each construct on business performance, as reported in the SmartPLS 4 IPMA output, while performance scores reflect rescaled (0–100) latent variable means. Constructs were classified into ‘high’ and ‘low’ importance categories relative to the median total-effect value (0.065), with constructs above the median classified as high importance and those at or below it as low importance, among the seven improvement-target constructs – the five antecedents plus MAIQ and MAAL. DQ is excluded from this cut-off calculation as it functions as the core mediator through which all upstream effects flow rather than as an independent investment target, and is retained in the high-importance category on the basis of its total effect (0.558) far exceeding this threshold; BEU is reported separately as a pure moderator rather than classified by this cut-off. Constructs that emerge as both high importance and low performance represent the highest priorities for improvement efforts. Table 4 presents the IPMA results.

**Table 4:** IPMA results – target construct: Business performance.

Construct	Importance	Performance (0–100)	Priority Quadrant	Managerial Implication
IT Capability (ITC)	0.122	52.01	HIGH / LOW-PERF	Highest priority: invest in digital/accounting systems
MAI Quality (MAIQ)	0.239	57.81	HIGH / MED-PERF	Improve MA report accuracy, timeliness, relevance
MA Adoption Level (MAAL)	0.169	55.32	HIGH / MED-PERF	Broaden MA tool adoption (budgeting, balanced scorecard, variance)
Decision Quality (DQ)	0.558	59.64	HIGH / MED-PERF	Core mediator – all upstream investments flow here
Top Mgmt Support (TMS)	0.063	58.17	LOW / MED-PERF	Maintain active owner-manager engagement with MA
Market Competition (MCI)	0.065	63.72	LOW / HIGH-PERF	Already high; maintain, no further intensification
Org. Decentralisation (DEC)	0.050	59.00	LOW / MED-PERF	Adequate; moderate formalisation as firms grow
Firm Size (SIZE)	0.034	63.06	LOW / HIGH-PERF	Small effect; focus on quality over scale
BEU (Moderator)	0.068	55.24	LOW / MED-PERF	Pure moderator; manage environmental risk exposure

*Note: Quadrant labels give the importance level / performance level (PERF = performance; MED = moderate); performance levels are classified as low (< 55), moderate (55–60) or high (> 60) on the 0–100 scale. Importance = standardised total effect on BP (direct + indirect); Performance = rescaled latent variable mean score (0 = lowest, 100 = highest). Highest action priority (high-importance / low-performance quadrant). For BEU (pure moderator), Importance reflects total effect on BP rather than a standard IPMA importance weight; its value is reported for completeness only and does not carry the same managerial interpretation as the importance scores of the antecedent and mediating constructs. Performance scores reflect SmartPLS-computed latent variable scores rescaled to a 0–100 range, which may differ slightly from rescaled raw item means because latent scores are weighted by outer loadings during estimation. For DEC specifically, the latent variable score (Performance = 59.00) is lower than its raw item mean would suggest (≈62.2), as the lowest-loading indicator (DEC4, λ = 0.682) downweights the construct score relative to a simple item average. Researchers replicating these scores from the raw dataset should use SmartPLS-estimated latent variable scores rather than item means.*

Fig 3 presents the mapping of all constructs within this importance-performance plane. The highest priority construct within this study is ITC – an element with the fourth largest total effect upon business performance (importance value of 0.122), behind DQ (0.558), MAIQ (0.239) and MAAL (0.169), but with the lowest performance score (52.01 out of 100). MAIQ (importance = 0.239, performance = 57.81) and MAAL (importance = 0.169, performance = 55.32) feature high importance but only moderate levels of realised performance potential. The finding that DQ emerges as the dominant driver of business performance in the role of the key serial mediator is again reflected in its IPMA importance score of 0.558 – this captures both its direct (total) effect on business performance (BP), as well as its role as a full-chain mediator of BP. MCI and SIZE reveal high performance values (63.72 and 63.06, respectively) but modest importance – confirming these elements as already well-leveraged within operations. They are thus not primary targets for improvement efforts.

**Fig 3:**
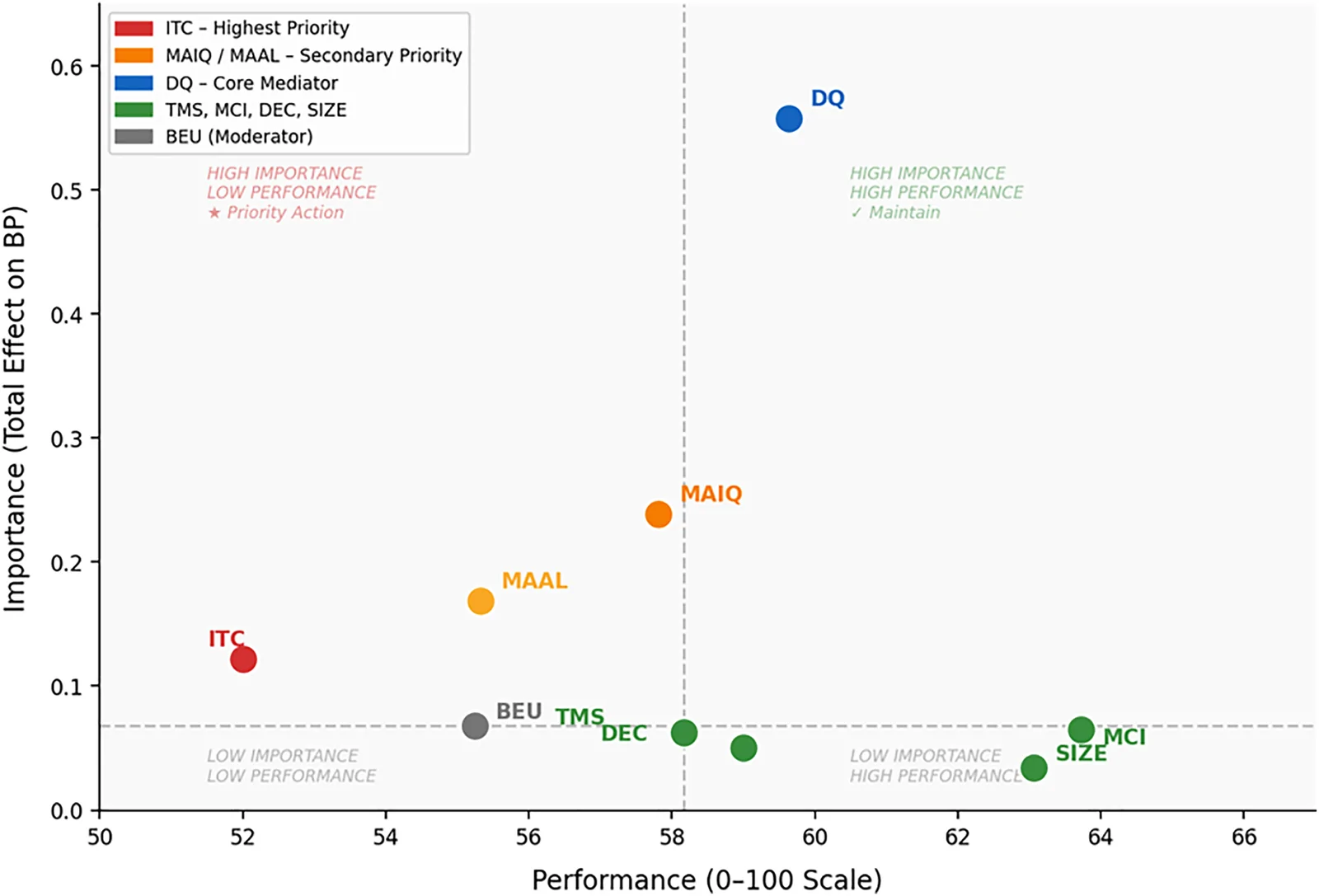
Importance-performance map analysis.

## 6. Discussion

### 6.1 Theoretical contributions

This investigation makes three main theoretical contributions to the literature upon management accounting and SMEs. First, it presents one of the first empirical tests of such a comprehensive model of serial mediation within the Vietnamese SME context – validating and extending findings of Contingency Theory predictions. The finding that each of the contextual antecedents exerts a statistically significant indirect effect on business performance through both the management accounting systems and the quality of decisions is confirmatory of the views of both Otley [17] and Chenhall [5] – a pattern that, to our knowledge, has rarely been tested empirically in this context. Such a finding helps to meet the calls for even more complete models within the management accounting research literature [7,13]. That all ten hypotheses are supported within the present study reflects a strong theoretical grounding of the model – rather than an absence of boundary conditions. Three observations support this interpretation. Each hypothesis was derived a priori from well-established theoretical frameworks – Contingency Theory and Information Processing Theory – rather than post-hoc to fit the data. The non-significance of the direct path from BEU to BP (β = 0.068, p = 0.088) shows that the model does not provide uniform support to every tested relationship – which is precisely as expected given the role of BEU as purely a moderator variable. The magnitudes of the supported paths vary substantially – from small (SIZE → MAAL, f^2^ = 0.053) to large (ITC → MAIQ, f^2^ = 0.397). While the limitations section acknowledges this finding, the single country, cross-sectional research design and use of self-report measurements mean that the magnitudes of these effects should be interpreted as specific to the context rather than universal.

Second, the study contributes to the extension of Information Processing Theory to the SME context and management accounting applications. From an Information Processing Theory perspective, the independent predictive power of MAIQ (β = 0.429) and MAAL (β = 0.303) over DQ confirms that quality and breadth of MA systems constitute distinct and complementary information processing channels – neither alone is sufficient, and both are necessary for optimal managerial decision quality.

Third, the finding of BEU moderation of BP (H9, β = 0.238) provides empirical support for the propositions of Contingency Theory within a previously under-examined context. The non-significance of the path from BEU to BP confirms that environmental uncertainty functions as a pure moderator within this model – a finding that helps to specify the conditions under which system quality and decision competence produce the largest returns in relation to performance. Such a result is in accord with both Gomez-Conde et al. [21] and Scaliza et al. [23], adding Vietnamese SME evidence to the growing body of research on contingency within emerging economies. This finding is consistent with recent PLOS ONE evidence from Vietnamese manufacturing firms showing that environmental pressures and uncertainty modulate the pathway between organisational capabilities and performance, reinforcing the position that contingency variables are essential rather than optional in emerging economy management accounting models [34].

### 6.2 Interpretation of specific hypothesis findings

The effect of ITC upon MAIQ established itself as the dominant relationship (β = 0.508) compared to that of TMS leading to MAIQ (β = 0.263). The findings indicate that within Vietnamese SMEs whose accounting systems remain paper-based or only employ basic bookkeeping software, no degree of top management commitment will lead to the production of timely and comprehensive management accounting reports without first securing adequate digital infrastructure. This finding contrasts with that common within developed economy studies [24], indicating that investment in digital infrastructure must precede any effort to develop leadership commitment within the Vietnamese SME context.

The relationship between MCI and MAAL emerged as the largest among the three antecedents to MAAL – in keeping with Gordon and Miller’s [16] classic contingency argument. This finding has been confirmed both by Bui et al. [3] and Ylä-Kujala et al. [53] across different SME contexts. Such findings suggest that policy interventions may be more effective if they frame adoption of management accounting systems in terms of competitive intelligence and benchmarking benefits rather than regulatory compliance.

The effect of DQ upon BP emerged as the strongest direct path within the model – confirming that decision quality is the pivotal element linking management accounting investment to business performance. While previous studies have all but certainly implied this relationship [8,22], the present study measured such an outcome directly. The confirmation of DQ both directly and with high statistical power (t = 18.88) implies that even modest improvements to decision quality arising from development of management accounting systems will lead to substantial returns in performance outcomes. This finding also resonates with findings within both Mittelstand and family-influenced SME contexts [54] – in each case, the quality of managerial decision-making processes, especially those involving financial managers, was shown to mediate the relationship between organisational capabilities and performance outcomes.

### 6.3 IPMA implications for practice

The findings of the IPMA study help to translate statistical results into a practical agenda for resource allocation. The position of ITC within the high-importance but low-performance quadrant (Performance = 52.01) indicates that investment within digital accounting infrastructure represents the single highest-return intervention for Vietnamese SMEs. Such development would encompass moving from basic ledger software platforms to integrated cloud-based offerings, as well as digitising cost tracking systems – an intervention that government digitalisation programmes should prioritise. Both MAIQ (importance = 0.239, performance = 57.81) and MAAL (importance = 0.169, performance = 55.32) score highly for importance relative to the antecedents but show only moderate levels of current performance realisation – confirming a substantial potential for performance growth through investment in both accounting system quality and breadth. The modest importance of TMS (0.063) relative to ITC (0.122) again indicates that infrastructure issues are the binding constraint upon development of management accounting systems within Vietnamese SMEs.

## 7. Conclusion

This study presents one of the first comprehensive tests of a model of serial mediation relating both organisational and technological antecedents – top management support, IT capability, decentralisation of authority within organisations, firm size and market competition intensity – to business performance outcomes, through both management accounting information quality and management accounting adoption levels, and through managerial decision quality. The uncertainty of the business environment has been confirmed as a positive moderating influence upon such a decision quality and performance outcome. Using PLS-SEM and IPMA on valid data collected from 503 managers of SMEs in Vietnam, the study has found support for each of the ten hypotheses – each with high statistical power.

Key findings indicate that information technology capability is the dominant antecedent to management accounting information quality (β = 0.508 vs. TMS’s β = 0.263); market competition intensity proves to be the strongest determinant of management accounting adoption (β = 0.385) relative to the other factors influencing adoption; both management accounting information quality (β = 0.429) and adoption level (β = 0.303) contribute independently to managerial decision quality, which in turn drives business performance (β = 0.558); business environment uncertainty proved a pure moderator (β = 0.238); all five serial indirect effects were statistically significant; and the IPMA analysis identifies information technology as the single highest-priority area for improvement efforts, with management accounting information quality and adoption as secondary priorities.

These findings have concrete implications for multiple stakeholder groups. SME owner-managers should focus upon upgrading digital capabilities prior to expanding their adoption of management accounting tools. Management accounting educators can benefit from incorporating digital accounting competencies into training programmes for SMEs. Government policy stakeholders thus have clear justifications for subsidising digital accounting tool adoption among such Vietnamese SMEs.

The study's scope may be interpreted within the following boundary conditions, each of which also defines avenues for future research. First, the cross-sectional design merits careful interpretation. Cross-sectional PLS-SEM is appropriate for explanatory and predictive research goals [37,38], and the present study is explicitly framed as such. Several design features attenuate the causal-inference limitation. The serial mediation chain is theoretically grounded in Contingency Theory [5,16,17] and Information Processing Theory [18], which establish the theoretical direction of effects prior to data collection — the antecedents are structural and technological characteristics of the firm, which logically precede information-system outcomes and managerial decision quality in time. The five serial mediation paths all yield bootstrapped confidence intervals excluding zero, and PLSpredict Q^2^ values confirm out-of-sample predictive relevance for every endogenous construct [46], demonstrating that the model's associations are not artefacts of the sample. Harman's single-factor test (28.2 per cent), full collinearity VIF assessment (all < 3.3) [48], and procedural separation of predictor and outcome items [49] collectively provide strong, convergent evidence that substantially mitigates concerns about common method bias as an alternative explanation, though – as acknowledged in Section 4.3 – such procedural and statistical remedies cannot eliminate this possibility entirely given the single-respondent, self-report nature of the DQ and BP measures. The consistency of effect directions with prior empirical evidence from comparable Vietnamese and ASEAN contexts [2,3] provides further convergent support. Notwithstanding these attenuating factors, temporal ordering of effects cannot be confirmed from cross-sectional data alone. Longitudinal panel designs or quasi-experimental studies exploiting variation in the timing of digital accounting investments would be the most direct means of establishing causal precedence and represent the highest-priority methodological extension of this work. Second, the single-country scope limits direct generalisability but this was a deliberate, theoretically justified choice. A single-country design controls for institutional heterogeneity — regulatory regime, accounting standards, language, and cultural norms — that would otherwise confound cross-national comparisons [5,6]. Vietnam is structurally representative of ASEAN transition economies more broadly: the SME sector's approximately 45 per cent contribution to GDP, the prevalence of founder-managed firms, co-existing digital modernisation mandates and legacy paper-based practices, and a rapid growth trajectory under international trade integration are structural characteristics shared with Indonesia, Thailand, the Philippines, and Cambodia. The model's theoretical foundations are explicitly context-neutral and have been validated across diverse national settings in prior contingency accounting research [5,27]. The most direct generalisability test would be multi-country replication across comparable ASEAN economies, treating institutional context as a between-group moderator in a multi-group analysis design. Third, the sample was drawn from the VCCI membership database and covers three major commercial centres, introducing a selection effect towards more formally organised SMEs; rural and provincial enterprises are excluded. The sample also skews towards small firms (90.3 per cent), limiting inference to this sub-category. Fourth, business performance and decision quality were measured with subjective, self-reported items reflecting managers’ own evaluations rather than objective or archival financial data, and all model constructs were assessed through a single informant per enterprise (typically a general director or owner-manager, or a finance manager). These are related but distinct sources of potential bias; the multi-layer set of common method bias tests substantially reduces this concern, but mixed-methods designs combining survey and archival financial data, and multi-informant designs, would further strengthen construct validity. Fifth, because the model does not include direct paths from the antecedents to decision quality or business performance, full and partial mediation cannot be distinguished; estimating these direct paths as a robustness check is a natural extension of this work. Finally, the R^2^ of 0.326 for decision quality indicates that antecedents beyond the five modelled here remain to be identified.

## Supporting information

S1 Appendix HTMT discriminant validity matrix. Heterotrait-monotrait (HTMT) ratios for all pairs of constructs; all off-diagonal values are below the 0.85 threshold.
(DOCX)

S2 Appendix Survey questionnaire. Full survey instrument used for data collection (respondent profile and measurement items).
(DOCX)
