# Supplementary material for: From accounting systems to business performance: A serial mediation model with IPMA evidence from Vietnamese SMEs
Source: PLoS One. 2026 Oct 27;21(10):e0361420. doi: 10.1371/journal.pone.0361420 (PMC13712740; doi:10.1371/journal.pone.0361420)
Supplement: Supplementary file 1 [file pone.0361420.s001.docx]

**S1 Appendix. HTMT discriminant validity matrix**

Reference numbers in square brackets refer to the reference list of the main manuscript.

**Table A1: Heterotrait-Monotrait (HTMT) Ratio Matrix - All Off-Diagonal Values Below 0.85 Threshold [51]**

|  | **TMS** | **ITC** | **DEC** | **MCI** | **MAIQ** | **MAAL** | **DQ** | **BP** | **BEU** |
| --- | --- | --- | --- | --- | --- | --- | --- | --- | --- |
| **TMS** | - |  |  |  |  |  |  |  |  |
| **ITC** | 0.40 | - |  |  |  |  |  |  |  |
| **DEC** | 0.62 | 0.28 | - |  |  |  |  |  |  |
| **MCI** | 0.45 | 0.32 | 0.53 | - |  |  |  |  |  |
| **MAIQ** | 0.54 | 0.72 | 0.31 | 0.26 | - |  |  |  |  |
| **MAAL** | 0.53 | 0.30 | 0.70 | 0.75 | 0.24 | - |  |  |  |
| **DQ** | 0.47 | 0.43 | 0.40 | 0.40 | 0.59 | 0.47 | - |  |  |
| **BP** | 0.38 | 0.42 | 0.39 | 0.41 | 0.53 | 0.40 | 0.73 | - |  |
| **BEU** | 0.48 | 0.59 | 0.47 | 0.40 | 0.34 | 0.44 | 0.35 | 0.36 | - |

*Note: All HTMT values < 0.85 threshold [51], confirming discriminant validity. Lower triangle shown. SIZE excluded as formative composite (HTMT criterion does not apply to formative constructs).*
