# Supplementary material for: From accounting systems to business performance: A serial mediation model with IPMA evidence from Vietnamese SMEs
Source: PLoS One. 2026 Oct 27;21(10):e0361420. doi: 10.1371/journal.pone.0361420 (PMC13712740; doi:10.1371/journal.pone.0361420)
Supplement: Supplementary file 2 [file pone.0361420.s002.docx]

**S2 Appendix. Survey questionnaire**

Reference numbers in square brackets refer to the reference list of the main manuscript.

SECTION A: RESPONDENT PROFILE - Please tick or fill in the most appropriate response.

A1. Role: General Director/Owner-Manager | Finance Manager | Operations Manager | Sales Manager | Other

A2-A3. Gender / Age: Male | Female | Prefer not to say; Under 30 | 30-39 | 40-49 | 50-59 | 60+

A4. Education: Secondary/Vocational | Diploma | Bachelor | Postgraduate

A5. Industry: Manufacturing | Trading/Retail | Services | Other

A6. Enterprise Size [36]: Micro (<10) | Small (10-199) | Medium (200-300)

A7. Employees (SIZE indicator 1): <10 | 10-49 | 50-99 | 100-199 | 200-300

A8. Annual Revenue/VND billion (SIZE indicator 2): <3 | 3-10 | 10-50 | 50-100 | >100

A9. Total Assets/VND billion (SIZE indicator 3): <3 | 3-10 | 10-50 | 50-100 | >100

A10. Years in Operation: <3 | 3-5 | 6-10 | 11-20 | >20 years

A11. Location: Ho Chi Minh City | Hanoi | Da Nang | Other

SECTION B: SURVEY ITEMS (1 = Strongly Disagree to 5 = Strongly Agree)

**B1. Top Management Support (TMS) [24]**

TMS1. Senior management actively encourages the use of formal management accounting information in decision-making.

TMS2. Senior management allocates sufficient financial resources for developing and maintaining our management accounting systems.

TMS3. Senior management trusts and regularly uses management accounting reports to guide strategic and operational decisions.

TMS4. Senior management demonstrates a clear commitment to improving the quality of accounting information available to managers.

**B2. IT Capability (ITC) [27,28]**

ITC1. Our enterprise uses accounting software or digital tools (e.g., MISA, FAST, cloud platforms) to manage financial and cost data.

ITC2. Our enterprise is capable of integrating data from different functional areas into unified accounting reports.

ITC3. Our IT systems allow us to generate management accounting reports in a timely manner.

ITC4. Our enterprise has the digital infrastructure necessary to support advanced management accounting analysis and performance monitoring.

**B3. Organisational Decentralisation (DEC) [30]**

DEC1. Subordinate managers have significant authority to make pricing decisions independently.

DEC2. Departmental managers can allocate budgets and resources without requiring approval for every expenditure.

DEC3. Managers at various levels have authority to respond to operational issues without referring everything upward.

DEC4. Our enterprise delegates strategic planning responsibilities to functional managers, not only to the owner-director.

**B4. Market Competition Intensity (MCI) [21]**

MCI1. Price competition in our industry is intense - competitors frequently cut prices to attract customers.

MCI2. New competitors enter our market regularly, making it difficult to maintain market share.

MCI3. Our customers have strong bargaining power and frequently demand lower prices or better terms.

MCI4. Substitute products or services represent a significant competitive threat to our enterprise.

**B5. MAI Quality (MAIQ) [10,22]**

MAIQ1. The management accounting information produced in our enterprise is accurate and reliable.

MAIQ2. Management accounting reports are delivered in a timely manner - information is available when managers need it.

MAIQ3. The management accounting information we receive is relevant to our specific operational and strategic decisions.

MAIQ4. Our MA system provides sufficiently comprehensive coverage of costs, revenues, and performance outcomes.

**B6. MA Adoption Level (MAAL) [9,13]**

MAAL1. Our enterprise uses formal budgeting processes (annual budgets, monthly budget tracking) as a standard management practice.

MAAL2. Our enterprise regularly conducts variance analysis - comparing actual costs and revenues against budgeted figures.

MAAL3. Our enterprise uses formal cost accounting methods (e.g., product costing, cost allocation) to inform pricing and operational decisions.

MAAL4. Our enterprise uses structured performance measurement tools (e.g., KPIs, balanced scorecard) to monitor performance.

**B7. Managerial Decision Quality (DQ) [8]**

DQ1. The decisions made by managers in our enterprise are well-informed - based on reliable data and thorough analysis.

DQ2. Managers make decisions in a timely manner - we rarely miss opportunities due to slow decision-making.

DQ3. Decisions are consistently aligned with our strategic objectives and business priorities.

DQ4. Overall, the quality of managerial decision-making in our enterprise is high - we make the right choices more often than not.

**B8. Business Performance (BP) [3,9]**

BP1. Compared to our key competitors, our enterprise has achieved strong revenue growth over the past two to three years.

BP2. Our enterprise has maintained satisfactory profitability over the past two to three years, relative to our industry peers.

BP3. Our enterprise has successfully expanded or maintained its market share over the past two to three years.

BP4. Overall, the operational efficiency of our enterprise compares favourably with similar firms in our industry.

**B9. Business Environment Uncertainty (BEU) [23]**

BEU1. The demand for our products or services is highly unpredictable - difficult to forecast customer purchasing patterns even in the short term.

BEU2. The regulatory and legal environment affecting our business changes frequently and unpredictably, making compliance planning difficult.

BEU3. Technological changes in our industry are rapid and difficult to anticipate, frequently disrupting our operations or competitive position.

BEU4. The competitive landscape in our industry is highly unpredictable - competitor moves, new entrants, and market shifts are difficult to forecast.

C1. (Optional) What is the single most important factor that would help your enterprise improve the quality and use of management accounting information?
